# Column-Parallel Correlated Multiple Sampling Circuits for CMOS Image Sensors and Their Noise Reduction Effects

**DOI:** 10.3390/s101009139

**Published:** 2010-10-12

**Authors:** Sungho Suh, Shinya Itoh, Satoshi Aoyama, Shoji Kawahito

**Affiliations:** 1 Research of Institute, Shizuoka University, 3-5-1 Johoku Nakaku Hmamatsu, Shizuoka, Japan; E-Mails: sito@idl.rie.shizuoka.ac.jp (S.I.); kawahito@idl.rie.shizuoka.ac.jp (S.K.); 2 Brookman Technology Inc., 3-1-7 Wajiyama Nakaku Hmamatsu, Shizuoka, Japan; E-Mail:saoyama@brookmantech.com (S.A.)

**Keywords:** CMOS image sensor, low noise, wide dynamic range, column-parallel correlated multiple sampling, folding integration technique

## Abstract

For low-noise complementary metal-oxide-semiconductor (CMOS) image sensors, the reduction of pixel source follower noises is becoming very important. Column-parallel high-gain readout circuits are useful for low-noise CMOS image sensors. This paper presents column-parallel high-gain signal readout circuits, correlated multiple sampling (CMS) circuits and their noise reduction effects. In the CMS, the gain of the noise cancelling is controlled by the number of samplings. It has a similar effect to that of an amplified CDS for the thermal noise but is a little more effective for 1/*f* and RTS noises. Two types of the CMS with simple integration and folding integration are proposed. In the folding integration, the output signal swing is suppressed by a negative feedback using a comparator and one-bit D-to-A converter. The CMS circuit using the folding integration technique allows to realize a very low-noise level while maintaining a wide dynamic range. The noise reduction effects of their circuits have been investigated with a noise analysis and an implementation of a 1Mpixel pinned photodiode CMOS image sensor. Using 16 samplings, dynamic range of 59.4 dB and noise level of 1.9 *e*^−^ for the simple integration CMS and 75 dB and 2.2 *e*^−^ for the folding integration CMS, respectively, are obtained.

## 1. Introduction

Complementary metal-oxide-semiconductor (CMOS) image sensors offer many advantages over CCDs’, such as system-on-chip, low power consumption and possibly lower cost of camera systems [1,2]. The pinned photodiode technology introduced to CMOS image sensors in mid-90s effectively reduces the dark current and cancels the *kTC* noise of pixels, and attains high conversion gain [3]. As a result, the noise level of recent CMOS image sensors is becoming even better than CCD image sensors, especially for applications that high frame-rate is required. Low-noise high-gain column readout circuits used for pixel noise cancelling and signal sampling are greatly contributing to the reduction of the readout random noise [4–7]. This high-gain column amplifier reduces the noise of wideband amplifiers at the output of image sensors by a factor of the gain, and if the amplifier reset noise is cancelled, the thermal noise due to the pixel source follow (SF) amplifier can be reduced by a factor of square root of the gain [7]. Nevertheless, for next-generation low-noise CMOS image sensors, advanced noise reduction techniques are needed in order to more effectively reduce the pixel SF amplifier noises, especially 1/*f* and random telegraph signal (RTS) noises and maintain the signal dynamic range.

In this paper, the noise reduction effects of another type of column-parallel high-gain signal readout circuits, correlated multiple sampling (CMS) circuits, for CMOS image sensors are discussed. In the CMS, both reset and signal levels of pixel outputs are sampled for multiple times and summed up, and the difference of the average of the two levels is calculated for pixel-related noise cancelling. Two types of the CMS circuits are proposed. One is with a simple integration and the other is with a folding integration. In the folding integrator, the signal swing of the integrator output is suppressed by a negative feedback using a comparator (one-bit analog-to-digital converter (ADC)) and a one-bit digital-to-analog converter (DAC). This allows us to reduce the readout noise while maintaining the signal dynamic range. A prototype 1Mpixel CMOS image sensor with pinned photodiodes and the column-parallel CMS circuits has been implemented. The noise measurement results show an interesting behavior for low-noise pixels and noisy pixels. The noise behavior in low-noise pixels due to thermal and 1/*f* noises and noisy pixels due to RTS and RTS-like noises is discussed with a noise analysis using a transfer function of the CMS.

## 2. Column-Parallel Correlated Multiple Sampling Circuits

### 2.1. Simple Integration

**Figure 1.**
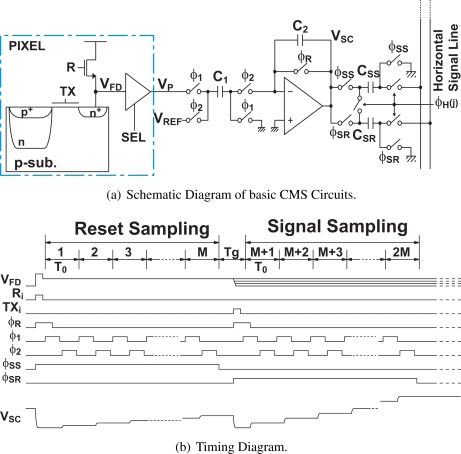
Schematic and Timing Diagrams of Implemented basic CMS Circuits.

Figure 1(a) shows the schematic diagram of a basic CMS circuit using a simple switched-capacitor (SC) integrator and two sample-and-hold (S/H) circuits. In the image array, a 4-transistor active pixel with a pinned photodiode is used. The timing diagram for the operation of the CMS is shown in Figure 1(b) where *T*_0_ is the sampling time, and *T_g_* is the interval of reset and signal samples.

**Figure 2.**
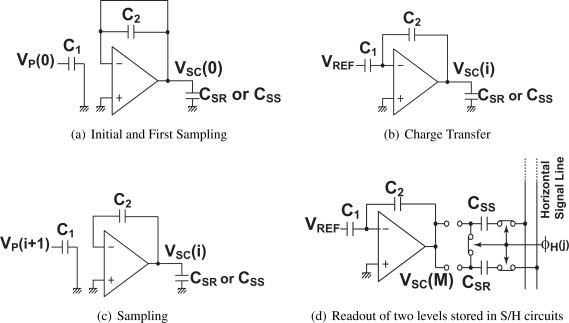
Phase Diagram of Correlated Multiple Sampling Circuits.

Figure 2 shows the phase diagram of the CMS circuits. As shown in Figure 2(a), before the CMS operation, the capacitor *C*_2_ is initalized by turning a switch controlled by *φ_R_* (shown in Figure 1(a)) on. In this phase, the charge stored in *C*_2_ due to the previous pixel signal is discharged to zero. During this phase, the first sampling of the reset level *V_P_*(1) is also performed by turning switches by *φ*_1_ on. In the next phase, the charge stored in *C*_1_ is transferred to *C*_2_ by turning switches by *φ*_2_ on as shown in Figure 2(b). The output for the first sampling *V_SC_*(1) is then given by

(1) $$V_{\mathrm{SC}}(1)=\frac{C_{1}}{C_{2}}(V_{P}(1)-V_{\mathrm{REF}}).$$

The second sampling is done by turning switches by *φ*_1_ on again while the charge due to the previous sampling is stored in *C*_2_ as shown in Figure 2(c). The charge stored in *C*_1_ is transferred to *C*_2_ with the circuit connection of Figure 2(b). The operation of Figure 2(b) and 2(c) is repeated for M times. The final output is sampled in *C_SR_*, and it is expressed as

(2) $$V_{\mathrm{SC}}(M)=\frac{C_{1}}{C_{2}}\sum_{i=1}^{M}(V_{P}(i)-V_{\mathrm{REF}}).$$

The same procedure is performed for the signal level and the final output with sampling M times is given by

(3) $$V_{\mathrm{SC}}(2M)=\frac{C_{1}}{C_{2}}\sum_{i=M+1}^{2M}\left(V_{P}(i)-V_{\mathrm{REF}}\right)$$

and the final output is sampled in *C_SS_*.

The two outputs stored in two S/H capacitors are scanned by a horizontal scanner and the difference is taken at the output using a differential charge amplifier for performing the pixel noise cancelling. The final differential output Δ*V_SC_* is then given by

(4) $$\Delta V_{\mathrm{SC}}=V_{\mathrm{SC}}(2M)-V_{\mathrm{SC}}(M)$$

(5) $$=\sum_{i=1}^{M}\left\{V_{P}(i)-V_{P}(i+M)\right\}$$

if *C*_1_ = *C*_2_. The first and second M samples are for reset and signal levels of the pixel output, respectively. Using the average of the reset and signal levels, $\bar{V_{\mathrm{PR}}}$ and $\bar{V_{\mathrm{PS}}}$, respectively, Δ*V_SC_* is given by

(6) $$\Delta V_{\mathrm{SC}}=M\left(\bar{V_{\mathrm{PR}}}-\bar{V_{\mathrm{PS}}}\right).$$

The simple integration CMS (SI-CMS) has a gain of M and a function of the difference of the average of the reset and signal levels of the pixel output.

### 2.2. Folding Integration Technique

The integration using multiple sampling is effective for the noise reduction of the pixel source follower [10]. However, the simple integration leads to the reduction of dynamic range. If the maximum signal swing at the SC integrator output is *V_SCM_*, the maximum input range is reduced to *V_SCM_* /*M* with sampling M times. To reduce the noise using multiple sampling while maintaining dynamic range, the folding integration technique is proposed.

**Figure 3.**
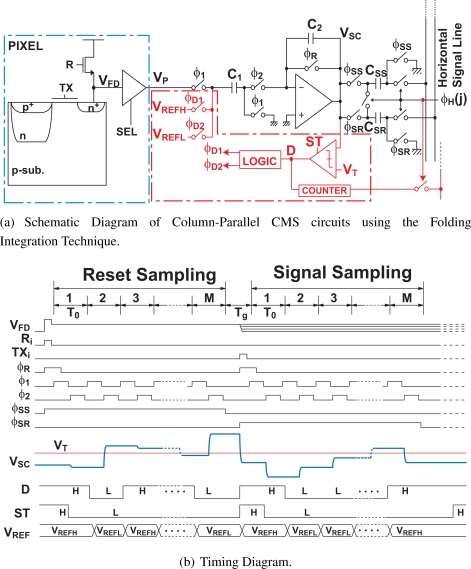
Schematic and Timing Diagram CMS circuits using the Folding Integration Technique.

Figure 3 shows the schematic and timing diagrams of the CMS circuits using the folding integration technique. In the folding integration CMS (FI-CMS) circuits, one comparator, two reference voltages *V_REFH_* and *V_ERFL_*, and control logics are added to the basic CMS circuits. The folding integration technique prevents the SC integrator output from causing saturation in such a way that the suitable reference voltage *V_REFH_* or *V_REFL_* is selected by the comparator output.

**Figure 4.**
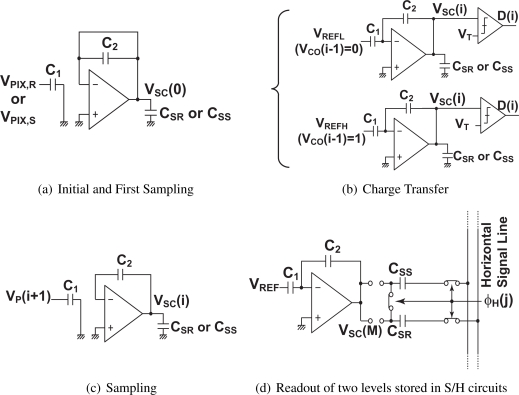
Phase Diagram of Correlated Multiple Sampling Circuits using Folding Integration Technique.

Figure 4 shows a phase diagram for the operation of the FI-CMS circuits. The initialization of feedback capacitor *C*_2_ and sampling the pixel output in the input capacitor *C*_1_ are done first as shown in Figure 4(a). In this phase, the initial value of the integrator output *V_SC_*(0) is set to 0 and the comparator output at the initial state *D*(0) is set to “1”. In the charge transfer phase shown in Figure 4(b), a physical bottom plate of *C*_1_ is connected either *V_REFH_* or *V_REFL_* to transfer charge in *C*_1_ to *C*_2_. Then the output of the SC integrator is compared with a threshold *V_T_*, and the comparator output of the *i*-th cycle *D*(*i*) (*i* ≥ 1) is given by

(7) $$D(i)=\{\begin{array}{cc} 0 & \left(\text{if}\;V_{\mathrm{SC}}(i)<V_{T}\right)\\ 1 & \left(\text{if}\;V_{\mathrm{SC}}(i)\geq V_{T}\right) \end{array}\;\;\;.$$

The SC integrator output of the *i*-th cycle (*i* ≥ 1) is expressed as

(8) $$V_{\mathrm{SC}}(i)=V_{\mathrm{SC}}(i-1)+\{\begin{array}{cc} V_{P}(i)-V_{\mathrm{REFH}} & \left(\text{if}\;D\;(i-1)=1\right)\\ V_{P}(i)-V_{\mathrm{REFL}} & \left(\text{if}\;D\;(i-1)=0\right) \end{array}$$

if *C*_1_ = *C*_2_. The process consisting of the pixel output sampling, charge transfer and comparison with the threshold is repeated M times. The final output is then given by

(9) $$V_{\mathrm{SC}}(M)=\sum_{i=1}^{M}V_{P}(i)-N_{1}V_{\mathrm{REFL}}-(M-N_{1})\;V_{\mathrm{REFH}}$$

where *N*_1_ is the number of counts that *D* takes “1”. The same procedure is applied to both reset and signal levels of the pixel outputs. Using the average of the reset and signal outputs, the difference of the SC integrator outputs, Δ*V_SC_* is given by

(10) $$\Delta V_{\mathrm{SC}}=M\left(\bar{V_{\mathrm{PR}}}-\bar{V_{\mathrm{PS}}}\right)-(N_{S}-N_{R})\;(V_{\mathrm{REFH}}-V_{\mathrm{REFL}})$$

where *N_R_* and *N_S_* are the number of counts that *D* takes “1” for the reset and signal levels, respectively.

**Figure 5.**
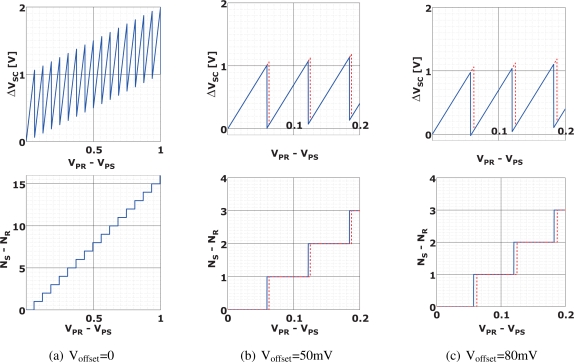
Characteristics of Integrated Outputs with Comparator Offset for M = 17 (Red dot-line: V_offset_ = 0).

Figure 5 shows the relationship between the input signal (*V_PR_* − *V_PS_*) and the analog output Δ*V_SC_* and the counter output (*N_S_* − *N_R_*) for M = 17 with and without the comparator offsets. A linear signal which corresponds to $M\times \left(\bar{V_{\mathrm{PR}}}-\bar{V_{\mathrm{PS}}}\right)$ in Equation (10) is reproduced in digital domain. The comparator offset may cause a non-linearity if Δ*V_SC_* exceeds the full scale range of the external A/D converter. Figure 5(b) and 5(c) show the cases that the comparator offsets are 50 mV and 80 mV, respectively. The A/D conversion of Δ*V_SC_* is supposed to be done by the full scale range of 0 to 2 V. The curve for Figure 5(c) exceeds the full scale range and the resulting reproduced signal have a non-linearity. However, in the case of Figure 5(b), the comparator offset does not cause the non-linearity. For instance, if *V_PR_* − *V_PS_* = 60 *mV* and M = 17, then *M* × (*V_PR_* − *V_PS_*) = 1020 *mV*. This can be expressed in two ways; (Δ*V_SC_*, *N_S_* − *N_R_*) = (1020 *mV*, 0), and (20 *mV*, 1). The both values reproduce the same *M* × (*V_PR_* − *V_PS_*) in digital domain. In ohter words, the comparator offset does not cause any error if Δ*V_SC_* is within the full scale range of the A/D conversion for Δ*V_SC_*.

As the input signal increases, the number of foldings increases and the resulting integrator output amplitude is compressed. The folding integrator reduces the thermal noise with the multiple sampling while maintaining the dynamic range. The both levels stored in S/H circuits are horizontally scanned and the difference of the two levels is taken at a differential charge amplifier at the output.

## 3. Noise Reduction Effects of Correlated Multiple Sampling Circuits

The noise reduction effect of the CMS circuits for thermal, 1/*f* and RTS noises can be calculated in frequency domain if the noise power spectrum and transfer function of the CMS operation are known. The transfer function of the CMS circuits as a discrete time system can be obtained from Equation (5). The interval of the two multiple samples *T_g_* influences to the 1/*f* noise reduction effect. For simplicity, *T_g_* is supposed to be integer multiple of the sampling time *T*_0_. Therefore, *T_g_* is given by *M_g_T*_0_, where *M_g_* is an integer. The final output of the CMS circuits as a function of discrete time, Δ*V_SC_*(*nT*_0_) is written as

(11) $$\Delta V_{\mathrm{SC}}(nT_{0})=\sum_{k=0}^{M-1}\left\{V_{P}((n-k)T_{0})-V_{P}((n-k-M-M_{g}+1)T_{0})\right\}$$

where *T*_0_ is the sampling period, *V_P_* ((*n* − *k*) *T*_0_) and *V_P_* ((*n* − *k* − *M* − *M_g_* + 1) *T*_0_) are reset and signal levels of pixel outputs of the *k*-th sample, respectively. The transfer function of the CMS is obtained with *z*-transform, and is expressed in the *z* domain as

(12) $$H_{\mathrm{CMS}}(z)=\frac{\left(1-z^{-M}\right)\;\;\left(1-z^{-(M+M_{g}-1)}\right)}{1-z^{-1}}.$$

If *M_g_* = 1, then it is simplified to

(13) $$H_{\mathrm{CMS}}(z)=\frac{{\left(1-z^{-M}\right)}^{2}}{1-z^{-1}}.$$

The output noise power, $\bar{\upsilon_{n,\mathrm{CMS}}^{2}}$ after the CMS process is calculated as

(14) $$\bar{\upsilon_{n,\mathrm{CMS}}^{2}}=\int_{0}^{\infty }S_{n}(f)\frac{1}{1+{\left(\omega /\omega_{c}\right)}^{2}}{\left|H_{\mathrm{CMS}}\left(e^{j\omega T_{0}}\right)\right|}^{2}\mathrm{df}$$

where *S_n_* (*f*) is a noise spectrum of the pixel source follower and *ω_c_* is the cut-off angular frequency of the sampling circuits in the CMS. The noise power spectrum of the pixel source follower is given by

(15) $$S_{n}(f)=S_{\mathrm{nt}}+\frac{k_{f}}{f}+\frac{k_{\mathrm{RTS}}\cdot \tau_{\mathrm{RTS}}}{1+{\left(2\pi \;f\tau_{\mathrm{RTS}}\right)}^{2}}$$

where *S_nt_* is the power spectrum density of the thermal noise, *k_f_* is the flicker noise coefficient, and *k_RTS_* and *τ_RTS_* are the RTS noise coefficient and relaxation time, respectively, of the RTS noise. The RTS noise due to a single trap has a Lorentzian-type spectrum as given by the third term of Equation (15), and *τ_RTS_* is given by

(16) $$\frac{1}{\tau_{\mathrm{RTS}}}=\frac{1}{\tau_{c}}+\frac{1}{\tau_{e}}$$

where *τ_c_* and *τ_e_* are mean time that the trap in the gate oxide captures and emits an electron, respectively [7].

The thermal noise with the CMS operation can be calculated without performing the integration of Equation (14) as

(17) $$\bar{\upsilon_{\mathrm{nt},\mathrm{CMS}}^{2}}=S_{\mathrm{nt}}\frac{\omega_{\mathrm{CMS}}}{2}$$

if *ω_CMS_* ≪ *ω_c_*, where *ω_CMS_* is the bandwidth (*cuf-off angular frequency*) of the CMS circuits given by

(18) $$\omega_{\mathrm{CMS}}=\frac{2}{T_{0}}\sin^{-1}\left(\frac{\sqrt{2}}{M}\right)≅\frac{2}{T_{0}}\frac{\sqrt{2}}{M}\;\;\;\;(for\;M\gg \;1).$$

**Figure 6.**
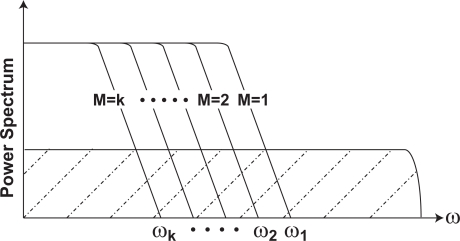
Bandwidth Reduction to Theraml Noise after CMS.

Therefore the thermal noise power is reduced in inverse proportion to M as shown in Figure 6 due to bandwidth limitation effect.

From Equation (12) with *z* = exp(*jωT*_0_), the noise power transfer function for the CMS is given by

(19) $${\left|H_{\mathrm{CMS}}\left(e^{j\omega T_{0}}\right)\right|}^{2}=\frac{4{\text{sin}}^{2}(\omega MT_{0}/2){\text{sin}}^{2}\{\omega \;(M+M_{g}-1)\;T_{0}/2\}}{{\text{sin}}^{2}(\omega T_{0}/2)}\;.$$

**Figure 7.**
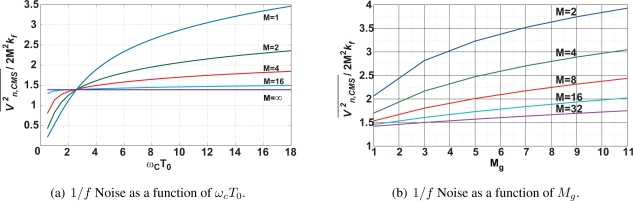
Noise Reduction Effect to 1/f Noises after CMS.

The 1/*f* noise power with the CMS operation can be calculated by Equation (14) and (19). The result as a function of *ω_c_T*_0_ is shown in Figure 7(a) for *M_g_* = 1 [10]. The noise power is normalized with 2*M*^2^*k_f_*. In the case of M = 1, the CMS is operating as the correlatd double sampling (CDS) [9]. For M = ∞, it approaches 1.39 which corresponds to a differential averager using continuous integration [7]. The noise power as a function of *M_g_* is shown in Figure 7(b). The noise power has a tendency to increase as *M_g_* increases. For effective noise reduction, *M_g_* should be minimized.

**Figure 8.**
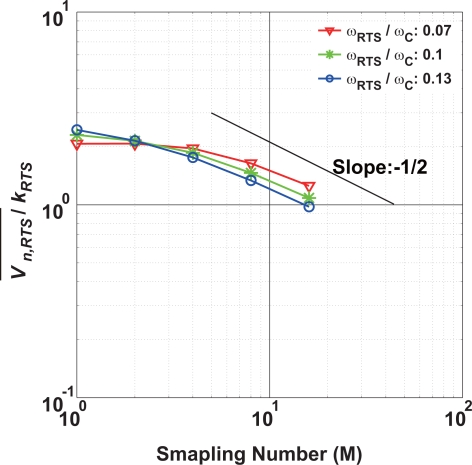
Estimated RTS Noise Amplitude as a function of M.

From Equation (14) and the third term of Equation (15), the normalized noise power with *k_RTS_* is given by

(20) $$\bar{\upsilon_{n,\mathrm{RTS}}^{2}}=\int_{0}^{\infty }\frac{k_{\mathrm{RTS}}/\omega_{\mathrm{RTS}}}{1+{\left(\omega /\omega_{\mathrm{RTS}}\right)}^{2}}\frac{1}{1+{\left(\omega /\omega_{c}\right)}^{2}}{\left|H_{\mathrm{CMS}}\left(e^{j\omega T_{0}}\right)\right|}^{2}df$$

where *ω_RTS_* = 1/*τ_RTS_*. The calculated RTS noise after the CMS process is shown in Figure 8. It shows the noise amplitude normalized by *k_RTS_* as a function of the number of samplings. Three curves for *ω_RTS_*/*ω_C_* = 0.07, 0.1 and 0.13 are plotted. If the bandwidth of the CMS, *ω_CMS_* determined by M is smaller than *ω_RTS_*, the noise reduction effect becomes efficient and the gradient of the noise amplitude to M approaches −1/2.

## 4. Measurement Results

**Figure 9.**
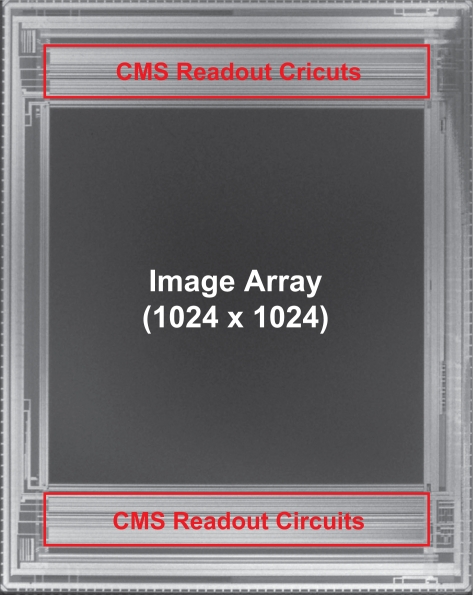
Implemeted chip photomicrograph.

A 1Mpixel CMOS image sensor with column-parallel FI-CMS circuits for the low-noise wide dynamic range readout is implemented with 0.18 *μm* CMOS technology with pinned photodiodes. The chip photomicrograph is shown in Figure 9. In this chip, the operation of the SI-CMS is also possible by disabling the function of folding. The pixel type is a 4-Tr pinned photodiode active pixel. The specifications of the CMOS image sensor are summarized in Table 1. The number of effective pixels is 1,024(H) × 1,024(V), the pixel size is 7.5 *μm* × 7.5 *μm*, and the conversion gain is 35 *μV*/*e*^−^. Chip outputs are come out by 4 channel output buffers, and are comprised of analog residue signals and counter output codes.

**Figure 10.**
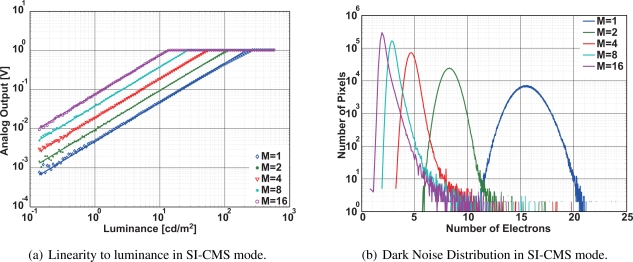
Characteristics in SI-CMS mode.

Figure 10(a) shows measurement results of the linearity of the implemented CMOS image sensor operating in the SI-CMS mode. Though the linearity is degraded at large output swing for M = 1, the gain of the signal in the linear region almost exactly follows the number of samplings. The dark noise distribution of the 1Mpixel CMOS image sensor is shown in Figure 10(b). The noise electron at the peak of distribution is about 15.6 *e*^−^ when the sampling number is 1. The noise electron is reduced to smaller than 2 *e*^−^ for the sampling number of 16. The noise distribution is entirely shifted to the lower noise as increasing the number of samplings.

**Figure 11.**
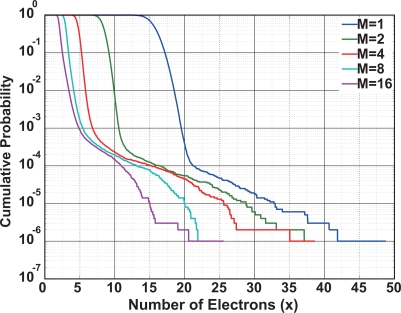
Cumulative Probability in SI-CMS mode.

Using the noise distribution shown in Figure 10(b), a cumulative probability (C.P.) *P*(*y*) of the temporal noise as a function of noise electrons given by

(21) $$P(y)=\frac{\sum_{x=y}^{\infty }h(x)}{\sum_{x=0}^{\infty }h(x)}$$

is calculated as shown in Figure 11, where *h*(*x*) is the distribution as a function of the noise electrons (*x*). The pixel noise sources are mainly thermal and 1/*f* noises of the source follower amplifier. However, some pixels have a large noise due to RTS and RTS-like noises. The tailing part of distribution of the cumulative probability (< 10^−4^) is due to the RTS- and RTS-like noise. Therefore, pixels are roughly classified into two types, low noise (C.P. > 10^−4^), and noisy pixels (C.P. < 10^−4^). In low noise region, the dominant noise components are the thermal and 1/*f* noises. In large noise region, the noisy pixels are mainly due to RTS or RTS-like noises [11]. The results of Figure 11 show that the CMS has a noise reduction effect of RTS and RTS-like noises if the number of samplings is increased.

**Figure 12.**
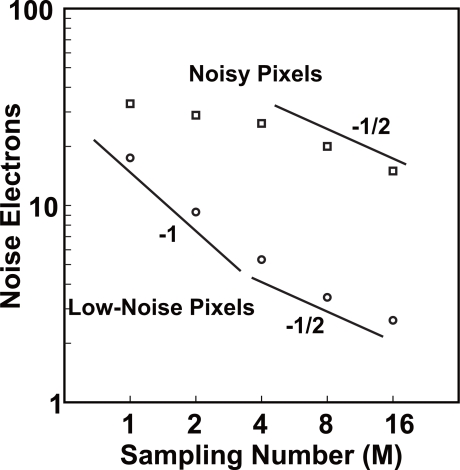
Temporal noise Charateristics for the sampling times of 1Mpixel CMOS imager.

Figure 12 shows behaviors of the low-noise and noisy pixels when the number of samplings is increased. Two plots show the values of noise electrons for low-noise and noisy pixels at 90 and 0.01% of cumulative probabilities, respectively. In the low-noise pixels, the noise amplitude is in inverse proportion to M, for *M* ≤ 4. This means that the dominant noise comes from the circuits and systems connected at the back of the CMS integrator such as output buffers and external ADCs. For M > 4, the CMS has a tendency of noise reduction in inverse proportion to square root of M. In this region, the dominant noise is due to thermal noise of the pixel source follower as predicted by Equations (17) and (18). Though it is not clearly shown in Figure 12, the noise reduction effect of the CMS is limited by the 1/*f* noise of the pixel source follower if the number of samplings is increased to larger than 16 as predicted by results of Figure 7(a). In the noisy pixels, the noise reduction effect of the CMS is very small for M ≤ 4, and the noise reduction factor ( $\Delta \bar{\upsilon_{n}}/\Delta M$) seems to approach to −1/2 for M > 4. This result could be explained by the results of Figure 8 if the dominant noise is the RTS noise and 0.001% of the pixels (noisy pixels) takes *ω_RTS_/ω_C_* of around 0.1. However, further detailed measurements and analysis are necessary to conclude it.

**Figure 13.**
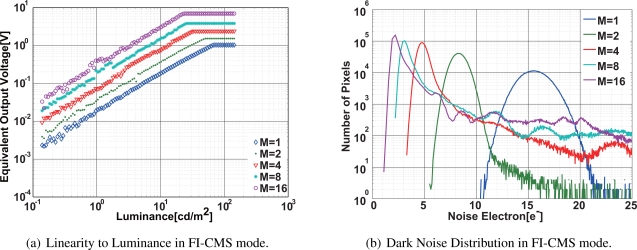
Characteristics in FI-CMS mode.

Figure 13(a) and 13(b) show characteristics of the linearity and the dark noise distribution with the 1Mpixel CMOS image sensor using the column-parallel FI-CMS, respectively. In the linearity, the equivalent output signal $M\times \left(\bar{V_{\mathrm{PR}}}-\bar{V_{\mathrm{PS}}}\right)$ is calculated in digital domain using the value of (*N_S_* − *N_R_*), and Δ*V_SC_* of Equation (10). The gain of the signal in the linear region follows the number of samplings. An important difference compared with the linear measurement results of SI-CMS (Figure 10(a)) is that the equivalent output is not limited to the maximum output swing of the integrator even though the number of samplings is increased to 16 while the output of the SI-CMS is limited to the maximum output swing of about 1 V. The maximum equivalent output swing of the FI-CMS is 6.94 V using M = 16 and the power supply voltage of 3.3 V. This shows the effectiveness for the wide dynamic range of the FI-CMS.

The problem of the FI-CMS of the present design when compared with that of the SI-CMS is the degradation of linearity and longer tailing of the dark noise distribution, as shown in Figure 13. A possible reason of the degradation is a coupling of digital signal lines running along sensitive analog circuit nodes in the column. In the SI-CMS mode, these digital lines are always fixed to “0”. As shown in the noise distribution of Figure 13(b), the FI-CMS has a noise reduction effect of the multiple sampling similar to that of the SI-CMS. Theoretically, the FI-CMS has the same noise reduction effect as that of the SI-CMS. Since the pixel outputs have large offset deviations, the influence of the coupling noise depends on the pixel source follower offsets. In most of the pixels, the influence of the digital signal coupling noise is cancelled out by the CDS operation if the reset and signal samplings have the same operation. However, in some pixels, the CDS does not completely cancel the coupling noise due to the FI-CMS operation, and population of noisy pixel increases. Therefore, the tailing of the distribution in Figure 13(b) is due to large digital signal coupling noises. The majority of the noise distribution around the peak has a behavior similar to that of the SI-CMS. The mean value of the distribution is 2.2 electrons, which corresponds to the input-referred noise of 77 *μV_rms_*.

Table 2 shows a summary of measurement results of noise and dynamic range. In the case of the simple integration, the noise is reduced to smaller than 2 electrons while sacrificing the dynamic range to be 59.4 dB. On the other hand, in the case of the folding integration, the dynamic range of 75.0 dB (= 6.94 *V*/ (16 × 77 *μV*)) is attained while reducing the noise to 2.2 electrons.

## 5. Conclusions

In this paper, column-parallel correlated multiple sampling (CMS) circuits and their noise reduction effects have been investigated with an implemented 1Mpixel pinned photodiode CMOS image sensor. A simple integration CMS (SI-CMS) shows the effectiveness of system, thermal and RTS noise reductions. A very low noise level of less than 2 electrons is attained for sampling 16 times. A folding integraion CMS (FI-CMS) allows us to realize low-noise wide dynamic range CMOS image sensors. The dynamic range of 75 dB and noise level of 2.2 electrons have been achieved simultaneously. In the present design, the linearity and noise characteristics of the SI-CMS are better than those of the FI-CMS. Also, the SI-CMS which is simplified from the FI-CMS can be implemented in a smaller area at the column. Therefore the SI-CMS is useful if the dynamic range is not a great concern. To attain full advantage of the FI-CMS, improvements of the FI-CMS are necessary for further increasing dynamic range, improving the linearity, and reducing the noise level by redesigning the column readout circuits. A technique for reducing digital coupling noise proposed in [12] is useful.

## Figures and Tables

**Table 1.** Summary of 1Mpixel Pinned Photodiode CMOS Image Sensor.

Parameter	Value
Technology	0.18 *μm* 1P4M CIS Process
Chip Size	10(H) *mm ×* 125(V) *mm*
Number of Effective Pixels	1,024(H) *×* 1,024(V)
Pixel Type	4-Tr Pinned Photodiode APS
Pixel Size	7.5 *μm ×* 7.5 *μm*
Fill Factor	47.7% (w/o Micro Lens)
Pixel Output Range	1 V
Conversion Gain	35 *μV/e^−^*
Random Noise (SI-CMS)	15.7 *e^−^* (@M:1) / 2.1 *e^−^* (@M:16)
Median Noise (SI-CMS)	35.3 *e^−^* (@M:1) / 18.1 *e^−^* (@M:16)
Column FPN (SI-CMS)	27.9 *μV_rms_*
Frame Rate	10 fps
Power	1.8/3.3 V (Digital), 3.3 V (Analog)
Power Consumption	88.3 mW

**Table 2.** Noise and dynamic range of the SI- and FI-CMS.

	Simple Integration		Folding Integration	
Sampling Time	1	16	1	16
Noise Electron [*e^−^*]	15.6	1.9	15.5	2.2
Noise Voltage [*μV*]	546.4	66.9	543.2	77
Maximum Output [*V*]	1.0	1.0	1.0	6.94
Dynamic Range [*dB*]	65.3	59.4	65.3	75.0
